# Rotablation of a tricky calcified lesion in a tortuous right coronary artery

**DOI:** 10.1007/s12471-019-01364-2

**Published:** 2020-01-17

**Authors:** L. Rijk, S. Hoseyni Guyomi, J. J. Remmen, A. J. M. Oude Ophuis

**Affiliations:** Department of Cardiology, Canisius-Wilhelmina Ziekenhuis, Nijmegen, The Netherlands

## Answer

**Fig. 1:**
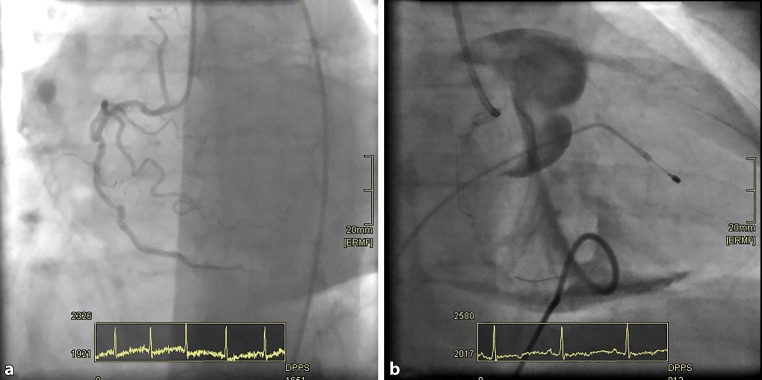
**a** Tortuous and calcified right coronary artery; **b** Angiographic image after rotablation

During initial angiography severely calcified lesions were observed (Fig. 1a) for which we scheduled a rotablation procedure. The set-up for this procedure included a venous sheath with access through the right femoral vein with pacemaker lead which can be seen in Fig. 1b.

Immediately after rotablation a perforation occurred in a mid-segment of the right coronary artery. The Rotawire was replaced by a support wire and the resulting occlusion was crossed, followed by placement of a 3.0 mm covered stent through a Guidezilla catheter.

Under echocardiographic guidance a pigtail catheter was inserted for pericardial drainage. The pigtail was flushed with contrast fluid to secure position of the drain. Contrast fluid showed to be contained within the pericardial cavity, indicating a correct drain position (Fig. 1b). The pericardial drain was left in situ after complete drainage, therefore the risk of occlusion of the drain was negligible. Furthermore, because of the position of the aortic valve and its connection with the pericardial cavity, aortic valve cusps can be seen (Fig. 1b).

The patient was treated with fluid resuscitation and inotropic support. The total pericardial drainage was 200 cc and the patient remained haemodynamically stable throughout the entire procedure. The procedure was continued through noncompliant balloon dilatation over the entire course of the vessel, followed by deployment of the 2.5 and 2.25 mm drug-eluting stent distally. Finally, a 3.5 mm covered stent was placed in the mid-segment.

Post deployment angiography revealed a covered perforation with thrombolysis in myocardial infarction (TIMI) 3 flow. Our patient was discharged in good clinical condition after 3 days of hospitalisation.

